# The prevalence of sexual dysfunction and its contributors among the women with multiple sclerosis

**DOI:** 10.1186/s12905-025-03653-y

**Published:** 2025-03-21

**Authors:** Hossein Mozhdehipanah, Negin Sharifi, Ali Emami, Mojgan Agha Abbaslou, Monirsadat Mirzadeh

**Affiliations:** 1 Department of Neurology, Bou Ali Sina Hospital, Qazvin University of Medical Sciences, Qazvin, Iran; 2 Department of General Psychiatry, Rhein Mosel Fachklinik, Andernach, Germany; 3 Qazvin University of Medical Sciences, Qazvin, Iran; 4 Department of Neurology, School of Medicine, Qazvin University of Medical Sciences, Qazvin, Iran; 5 Metabolic Diseases Research Center, Research Institute for Prevention of Non-Communicable Diseases, Qazvin University of Medical Sciences, Qazvin, Iran

**Keywords:** Sexual dysfunction, Multiple sclerosis, Quality of life

## Abstract

**Background:**

Sexual dysfunction is one of the main complaints in women with Multiple Sclerosis (MS) and it affects different aspects of their lives. We aimed to determine the prevalence of sexual dysfunction among MS patients and address the main contributing factors.

**Methods:**

This cross-sectional study was performed on 100 women with MS who were referred to Bu-Ali Sina Hospital in 2020–2021. We completed a 19-item Female Sexual Function Index (FSFI) questionnaire for each participant. We also collected demographic data. Patients were categorized based on their disability index Expanded Disability Status Scale (EDSS) by a neurology specialist. Data were analyzed using SPSS 25 software. A significance level of less than 0.05 was considered.

**Results:**

The mean age of study participants was 37.4 ± 7.8 years. We observed a negative significant correlation between EDSS and FSFI score (*r*=-0.371, P-value = 0.001). The correlation between FSFI score and age (*r*= -0.371, P-value = 0.001), parity (*r*= -0.322, P-value = 0.001), and disease duration (*r*= -0.371, P-value < 0.001) was also statistically significant. The mean of FSFI in the patients with university graduation was 25.0 ± 5.3, while it was 22.2 ± 6.4 and 20.1 ± 8.0 in diploma and less than diploma groups, respectively (P-value < 0.05). In linear regression, age, parity, length of disease and education level (just less than diploma and university graduated) as independent variables had a significant relationship with FSFI score (*P* < 0.05).

**Conclusion:**

The prevalence of sexual dysfunction among Iranian women who were suffering from MS was pretty high. Older age groups, less educated, and more disabled patients were addressed as more at-risk groups of impaired sexual function.

## Introduction

Multiple Sclerosis is an auto-immune disease in which the nerve fibers are attacked by the immune system and are demyelinated [1]. As a result, the speed of nervous control is decreased and consequently, it leads to permanent disability of the central nervous system [1]. Over 2.5 million people suffer from MS around the world and the number of patients is increasing [2]. This is also known as a health challenge in Iran where we have more than 75,000 MS cases [3].

Multiple Sclerosis is a multi-factorial disease and a wide range of risk factors including genetic agents, tobacco smoking, obesity, infectious agents, and geographical location play a role in its etiology [4, 5]. MS is a progressive and disabling disease that affects patients, their families, and even societies in different aspects of life [6].

Sexual dysfunction is one of the major complaints of MS patients. There is strong evidence that more than half of MS patients are not satisfied with their sexual function or even stopped having sex [7]. The prevalence of sexual dysfunction is pretty high and it is estimated almost 5 times higher than normal people [8, 9]. This disease could affect sexual desire, function, and even sexual position and these effects could be either direct (Primary sexual dysfunction) or indirect (Secondary/tertiary sexual dysfunction) [10–13]. Previous studies in Iran have reported the prevalence of sexual dysfunction among Iranian MS patients at 81.9%. Orgasm problems, spasticity, and sexual partner satisfaction were the most common complaints reported by Iranian MS patients [14].

Sexual dysfunction could affect the burden of disease by decreasing the quality of life of patients with MS. It could also impose a huge financial burden on patients, families, and communities. The reasons for excluding men were the higher prevalence of MS in women (female-to-male ratio of 3:1), sex-specific physiological differences affecting sexual dysfunction, and the need to reduce heterogeneity for a focused analysis. Additionally, including males would have required a larger sample size to maintain statistical power, which was beyond the scope of this study [15, 16]. However, to the best of our knowledge, few studies have been already performed regarding the prevalence of sexual dysfunction in MS patients in Iran and its associated factors. In the current study, we aimed to draw a better understanding of sexual dysfunction prevalence in MS patients in Iran and address the main contributor in this regard. Such information will help us to determine more at-risk groups and design more effective therapeutic and preventive strategies.

## Method

We performed an analytic cross-sectional study on 100 women with MS referred to the Bu-Ali hospital clinic in 2020–2021. We applied non-random convenience sampling on patients who had a willingness to participate in the study. Being married and definitive diagnosis of MS based on McDonald’s definition were the inclusion criteria. We also excluded patients with previously impaired sexual function due to genital disorders, diabetes, cardiovascular diseases, history of surgery, and radiotherapy on the hip, and those patients who did not complete informed consent.

A 19-item FSFI questionnaire with 6 subdomains including desire (2 items), arousal (4 items), Lubrication (4 items), Orgasm (3 items), Satisfaction (3 items), and sexual pain (3 items) was completed for each participant. All the questions were on a Likert scale and patients received a point from 1 to 5 for each question. The FSFI total score was the sum of all 19 questions and the maximum score was 36. The validity and reliability of the questionnaire have been already proven in the previous study [17]. The Persian format of the questionnaire was also validated by Mohammadi et al. (2008) [18]. The cut-point for FSFI score was considered at 26 and patients with a score less than 26 were diagnosed as patients with Female Sexual Dysfunction (FDS) [19].

We also used the Expanded Disability Status Scale (EDSS) as a measure of physical disability in MS patients. The EDSS was first developed by John Kurtzke in 1983 and is used for quantifying disability in patients with MS. The EDSS score ranges from 0 to 10 in 0.5 increments. In this scale by increasing EDSS the level of disability is increased. Scoring was done by a neurologist. We also completed demographic and clinical questionnaires including data on age, age at disease onset, disease duration, number of parity, education, job, and residential area for each patient.

As descriptive statistics, we reported mean and standard deviation for continuous variables. We also reported categorical variables using frequency number and percent. We used an independent T-test and one-way ANOVA to assess the association between FSFI score and dichotomous variables. Moreover, we reported Pearson correlation coefficients to investigate the relationship between continuous variables and FSFI mean score. All statistical analysis was performed using Stata software version 14.1.

Additionally, we performed multiple linear regression to evaluate the associations between FSFI scores and independent variables, including age, disease duration, EDSS score, and demographic characteristics. Before conducting the regression analysis, we verified the assumptions for multiple linear regression. Multicollinearity was assessed using the Variance Inflation Factor (VIF), and all independent variables had VIF values below 10, indicating no multicollinearity concerns. Residual plots and Q-Q plots were used to check for normality and homoscedasticity of residuals. Linearity between independent and dependent variables was confirmed through scatterplots.

The study was reviewed and approved by the local Institutional Review Board. Moreover, written informed consents were obtained from all individuals for participation and publication, according to the Declaration of Helsinki.

## Results

The study was performed on 100 women with multiple sclerosis. The mean age (± SD) of study participants and mean age at disease onset were 37.4 (± 7.8) and 28.4 (± 7.6), respectively which means disease duration was around 9.0 years on average. Overall, 44% of patients were university graduates, and around two-thirds of them were homemakers. The proportion of urban dwellers was 97.0%. Average score of EDSS and number of parity was also reported at 2.6 (± 1.1) and 1.3 (± 1.1) (Table 1). The prevalence of female sexual dysfunction among the study participants was 80%.

**Table 1:** The demographic and clinical characteristics of patients variable

Variable	
**Age, Mean (SD)**	37.4 (7.8)
**Age at disease onset**, **Mean (SD)**	28.4 (7.6)
**Education level**, **N (%)**	
Less than diploma	19 (19.0%)
Diploma	37 (37.0%)
University graduated	44 (44.0%)
**Occupation**, **N (%)**	
Home-Maker	75 (75.0%)
Office clerk	17 (17.0%)
Self-Employed	4 (4.0%)
Retired	4 (4.0%)
**Residential area**, **N (%)**	
Urban	97 (97.0%)
Rural	3 (3.0%)
**Parity**, **Mean (SD)**	1.3 (1.1)
**Disease duration**, **Mean (SD)**	9.0 (5.5)
**EDSS score**, **Mean (SD)**	2.6 (1.1)

SD = Standard Deviation

In Table 2, we investigated the correlation between FSFI and EDSS scores. We observed a negative significant correlation between EDSS and FSFI score (*r*=-0.371, P-value = 0.001). The same pattern was also observed regarding a couple of subdomains including desire (*r*= -0.293, P-value = 0.003), Lubrication (*r*= -0.3.4, P-value = 0.002), and pain (*r*= -0.243, P-value = 0.015). The correlation between FSFI score and age (*r*= -0.371, P-value = 0.001), parity (*r*= -0.322, P-value = 0.001), and disease duration (*r*= -0.371, P-value < 0.001) was also statistically significant (Table 2).

**Table 2:** The correlation between the characteristics of patients and FSFI total score and its subdomains

	Desire		Arousal		Lubrication		Orgasm		Satisfaction		Pain		FSFI score	
Factor	*r*	*p*	*r*	*p*	*r*	*p*	*r*	*p*	*r*	*p*	*r*	*p*	*r*	*p*
Age	-0.38	< 0.001	-0.36	< 0.001	-0.34	0.002	-0.25	0.01	-0.16	0.11	-0.24	0.01	-0.37	< 0.001
Parity	-0.49	< 0.001	-0.41	< 0.001	-0.23	0.01	-0.2	0.04	-0.13	0.17	-0.08	0.41	-0.32	0.001
Length of disease	-0.25	0.01	-0.19	0.05	-0.3	0.02	-0.2	0.11	-0.16	0.4	-0.24	0.03	-0.37	0.01
EDSS score	-0.23	0.003	-0.05	0.59	-0.3	0.002	-0.2	0.01	-0.16	0.11	-0.24	0.01	-0.37	< 0.001

We also assessed the average score of FSFI over categorical variables including residential area and education level. According to Table 3, no statistically significant difference was observed between rural and urban dwellers in terms of FSFI score and its subdomains. FSFI mean score in the patients with university graduation was 25.0 (± 5.3), while it was 22.2 (± 6.4) and 20.1 (± 8.0) in diploma and less than diploma groups, respectively and the observed difference was statistically significant (P-value = 0.013). The same difference was also observed in desire, arousal, and lubrication subdomains, and in all of them, the reported values for patients with university graduation were pretty high (P-value < 0.05) (Table 3).

**Table 3:** The mean score of FSFI and its subdomains by residential area and education level

Variable	Desire		Arousal		Lubrication		Orgasm		Satisfaction		Pain		FSFI	
Residential area	Mean	*p*	Mean	*p*	Mean	*p*	Mean	*p*	Mean	*p*	Mean	*p*	Mean	*p*
Urban	3.6		3.7		4		3.8		3.8		4		23.1	
Rural	3.4	0.78	3.7	0.98	3.6	0.53	3.6	0.82	3.6	0.77	3.4	0.51	21.3	0.64
**Education level**														
Less than diploma	2.8		3		3.6		3.2		3.6		3.7		20.1	
Diploma	3.2		3.4		3.8		3.7		3.9		4		22.2	
University graduated	4.2	0.001	4.1	0.003	4.4	0.03	4.1	0.18	3.9	0.82	4.1	0.52	25	0.01

The results of linear regression analysis showed that age, parity, length of disease and education level (just less than diploma and university graduated) as independent variables had a significant relationship with FSFI score (*P* < 0.05). In the multivariate model, only age and university graduated had a significant relationship with FSFI score. With the addition of one year, FSFI score decreased by 0.41 and university graduated could lead to an increase the FSFI score by 3.13 (*P* < 0.05, R^2^ = 0.22). (Table 4).

**Table 4:** The linear regression analysis for prediction of sexual function according to demographic factors and EDSS score

Variables	Unadjusted			Adjusted		
	B	Beta	*P* value	B	Beta	*P* value
Age	-0.31	-0.37	0.001	-0.41	-0.49	0.003
parity	-1.96	-0.33	0.001	0.09	0.01	0.9
Length of disease	-0.17	-0.2	0.04	0.14	0.16	0.26
EDSS score	-0.99	-0.17	0.09	-0.06	-0.01	0.91
Less than diploma	-3.59	-0.21	0.03	-1.27	-0.07	0.46
Diploma	-1.32	-0.98	0.32	-	-	-
University graduated	3.5	0.26	0.007	3.13	0.23	0.03

## Discussion

In the current study, we aimed to assess the prevalence of sexual dysfunction in Iranian women with MS disease who were referred to the Bu-Ali hospital clinic in Qazvin in 2020–2021. We found that 80% of our study participants were diagnosed with FDS which was in accordance with a previously published study by Dehghan et al. [14]. However, the reported prevalence by Ashtari et al. (63.5%) was considerably lower [20]. We also reported a higher prevalence of FDS in comparison to studies performed in more developed countries such as Italy (49.6%) [21], Poland (27.3%) [22] and Britain (21.4%) [23]. Although some part of this difference could be attributed to differences in the inclusion criteria, sampling method, or data collection tools, it seems the prevalence of sexual dysfunction in women with MS is more major problem in our country. One reason to explain this difference could be a result of cultural issues and a lack of public awareness about sexual issues [24]. Sexual dysfunction of women is considered a social stigma among Muslim women [24]. Besides, there are not enough public education and awareness programs regarding sexual issues in Iran and in such circumstances, women with sexual dysfunction are less likely to receive appropriate treatments [25].

We observed a statistically significant negative correlation between EDSS and FSFI mean score implying that increasing EDSS score leads to a decrease in sexual function. The observed inverse correlation in four subdomains including desire, lubrication, Orgasm, and pain was also significant. The inverse correlation between EDSS and FSFI scores observed in our study aligns with findings from several previous studies, which highlight the mechanisms underlying this relationship. Increased EDSS scores, indicative of greater physical disability, are associated with impairments in mobility, muscle strength, and coordination, all of which can directly impact sexual function. Additionally, higher EDSS scores often reflect more severe neurological damage, which can disrupt the neural pathways involved in sexual response, including arousal, lubrication, and orgasm. Those studies also emphasized the role of secondary factors such as fatigue, spasticity, and depression, which are more prevalent in patients with higher EDSS scores and can further exacerbate sexual dysfunction. These findings underscore the multifactorial nature of sexual dysfunction in MS patients and the importance of addressing both primary neurological and secondary psychosocial factors in clinical care [17, 21].

According to our findings, age was the other major contributor to FDS. Age was negatively correlated to FSFI score and all its subdomains which means in elder people all domains of sexual function including desire, arousal, lubrication, orgasm, satisfaction, and pain are impaired or declined. These findings were the side of previously published research by Konstantindis et al. and Gava et al. [17, 21]. Other studies have reported the same negative significant correlation between age and sexual functions in women with MS [11, 14].

A lack of public education and awareness programs regarding sexual health in Iran contributes significantly to the underdiagnosis and undertreatment of sexual dysfunction, particularly among women with MS. Cultural stigma surrounding discussions of sexual health often discourages women from seeking help, leading to a lack of recognition and treatment for their condition [26]. Studies have highlighted that healthcare providers in conservative societies may also feel unprepared or reluctant to address sexual health concerns, further exacerbating the issue [26, 27]. Additionally, limited access to specialized sexual health services and insufficient training of healthcare professionals in diagnosing and managing sexual dysfunction contribute to the problem. Addressing these barriers through targeted education programs, increasing public awareness, and integrating sexual health training into medical curricula could help reduce stigma and improve access to care for women with MS experiencing sexual dysfunction [27].

The reverse correlation was also reported between disease duration and parity with the FSFI mean score. These findings were from previous studies. Prolonged disease is associated with more progression and it increases the level of disability among MS patients. As we already discussed more disability can affect sexual function and it seems prolonged disease can increase SDF through this pathway.

Our study is among the few that have evaluated the contributing factors of sexual dysfunction among women with MS in Iran. Previous studies, such as those by Dehghan et al. [14] and Ashtari et al. [20], have also investigated this topic, reporting prevalence rates of 80% and 63.5%, respectively. While our findings align with Dehghan et al., they indicate a higher prevalence compared to Ashtari et al., which could be attributed to differences in study design, inclusion criteria, and assessment tools. Additionally, our results contrast with studies conducted in more developed countries, such as those by Gava et al. in Italy (49.6%) [21], Bartnik et al. in Poland (27.3%) [22], and Burri et al. in Britain (21.4%) [23], further highlighting potential cultural, social, and healthcare disparities. By focusing on Iranian women with MS, our study provides valuable insights into the unique sociocultural and systemic factors influencing sexual dysfunction in this population, underscoring the need for targeted interventions and further comparative research.

Moreover, we have used valid questionnaires and tools and robust statistical methods. However, our findings must be interpreted in the context of its limitations. Self-reported and cross-sectional method of study was the main limitation of our study. Besides, we used a non-random sampling method among less severe patients and it can affect the generalizability of our findings.

## Conclusion

We spotted a high prevalence of sexual dysfunction among women who were suffering from MS. Older age groups, less educated, and more disabled patients were addressed as more at-risk groups of impaired sexual function and must be considered for both preventive and therapeutic strategies.

## Data Availability

No datasets were generated or analysed during the current study.

## Abbreviations

MS: Multiple sclerosis
FSFI: Female sexual function index
EDSS: Expanded disability status scale
FDS: Female sexual dysfunction
